# Molecular signatures of T-cell inhibition in HIV-1 infection

**DOI:** 10.1186/1742-4690-10-31

**Published:** 2013-03-20

**Authors:** Marie Larsson, Esaki M Shankar, Karlhans F Che, Alireza Saeidi, Rada Ellegård, Muttiah Barathan, Vijayakumar Velu, Adeeba Kamarulzaman

**Affiliations:** 1 Molecular Virology, Department of Clinical and Experimental Medicine, Linköping University, Linköping, 58 185, Sweden; 2 Tropical Infectious Disease Research and Education Center (TIDREC), Department of Medical Microbiology, Faculty of Medicine, University of Malaya, Lembah Pantai, Kuala Lumpur, 50603, Malaysia; 3 Institute for Environmental Medicine, Karolinska Institute, Solna, Stockholm, 17 177, Sweden; 4 Centre of Excellence for Research in AIDS (CERiA), Department of Medicine, Faculty of Medicine, University of Malaya, Lembah Pantai, Kuala Lumpur, 50603, Malaysia; 5 Department of Microbiology and Immunology, Emory Vaccine Center, Emory University, 954 Gatewood Road, Atlanta, GA, 30329, USA

**Keywords:** BLIMP-1, CTLA-4, FoxP3, HIV-1, T-cell inhibition, LAG-3, PD-1, TIM-3, 2B4, CD160

## Abstract

Cellular immune responses play a crucial role in the control of viral replication in HIV-infected individuals. However, the virus succeeds in exploiting the immune system to its advantage and therefore, the host ultimately fails to control the virus leading to development of terminal AIDS. The virus adopts numerous evasion mechanisms to hijack the host immune system. We and others recently described the expression of inhibitory molecules on T cells as a contributing factor for suboptimal T-cell responses in HIV infection both *in vitro* and *in vivo*. The expression of these molecules that negatively impacts the normal functions of the host immune armory and the underlying signaling pathways associated with their enhanced expression need to be discussed. Targets to restrain the expression of these molecular markers of immune inhibition is likely to contribute to development of therapeutic interventions that augment the functionality of host immune cells leading to improved immune control of HIV infection. In this review, we focus on the functions of inhibitory molecules that are expressed or secreted following HIV infection such as BTLA, CTLA-4, CD160, IDO, KLRG1, LAG-3, LILRB1, PD-1, TRAIL, TIM-3, and regulatory cytokines, and highlight their significance in immune inhibition. We also highlight the ensemble of transcriptional factors such as BATF, BLIMP-1/PRDM1, FoxP3, DTX1 and molecular pathways that facilitate the recruitment and differentiation of suppressor T cells in response to HIV infection.

## Review

### Introduction

Functional senescence of virus-specific T cells and progressive loss of naïve CD4^+^ and CD8^+^ T cells are features of HIV infection [1]. One effect HIV infection has, is to facilitate the expansion of suppressor T cells, which compromises HIV-specific CD4^+^ and CD8^+^ T cell responses by acting in a contact-dependent manner [2-5]. HIV infection can alter the survival rates and regenerative capacity of T cells [6]. A recent study also showed that HIV-infected T cells serve as migratory vehicles for viral dissemination [7] and therefore once infected may not contribute to viral clearance. Importantly**,** the impairment of effector T-cell immune functions in HIV infected individuals is reportedly multifactorial [8], and upregulation of negative costimulatory and secretory factors and impaired cytokine production in HIV-specific T cells and other immune cells is believed to facilitate rapid disease progression and eventual systemic immune dysfunction [9,10]. Hence, the expression of inhibitory molecules on T cells has been proposed as a contributing factor for the suboptimal T-cell responses seen in HIV infection [2-6].

### Unraveling the complexity of T-cell costimulation

**Figure 1:**
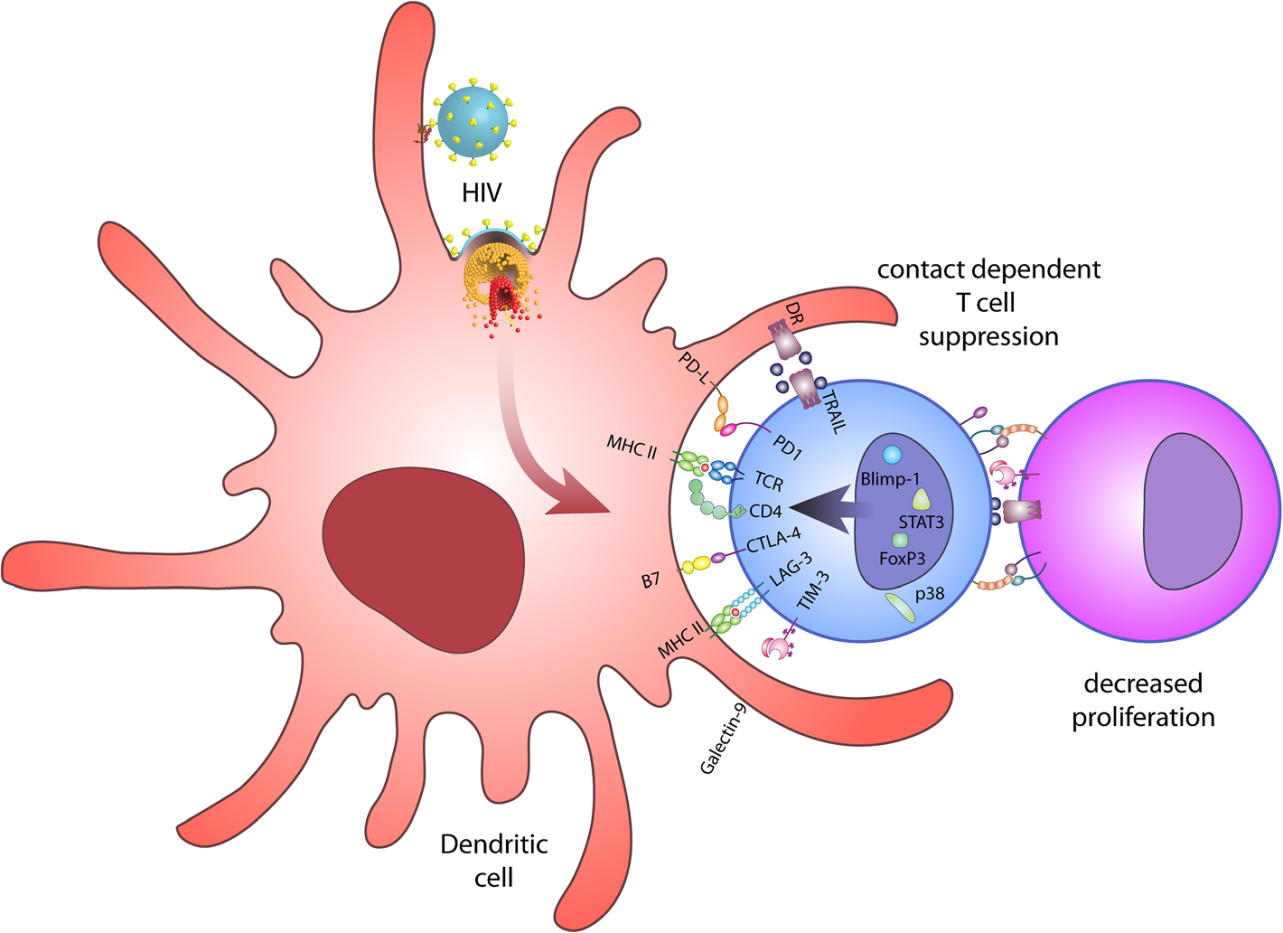
**Infection with HIV facilitates the upregulation of inhibitory molecules in T cells.** HIV-1 modulates host DCs to increase expression of numerous inhibitory molecules on expanded T cells. The expanded T cells are suppressor T cells [4] that act on other T cells present in the near vicinity in a contact-dependent manner [4], transforming them into suppressor cells and so contributing to eventual T-cell inhibition [3-5].

The first step of HIV-1 transmission is mucosal exposure and Langerhans cells lining the genital mucosa, constitute a front-line defense against invading virus [11,12]. These dendritic cells (DCs) pick up HIV-1 from mucosal sites, and migrate to peripheral lymph nodes to activate HIV-specific naïve T cells. During migration the DC changes its phenotype and increases the expression of maturation markers, e.g. CD83, MHC class I and II, costimulatory molecules, and lymph node homing molecules, e.g. CCR7 (CD197). These events are critical for efficient antigen presentation, downstream signaling, and T-cell activation [12]. The T cells play a key role in cell-mediated immune responses, and their activation is multifaceted and requires distinct signals. The first signal occurs when the TCR recognizes the antigenic peptide bound to MHC molecules on APCs. The second signal, the costimulatory signal, can either be positive or negative, the former necessary for achieving full T cell activation and initiation of effective immunity and the latter for the establishment and maintenance of peripheral tolerance, and abortive T-cell responses [13]. A balance between positive and negative costimulatory pathways is required to sustain a normal protective response and these pathways are therefore attractive therapeutic targets for chronic diseases associated with immune suppression. The surface receptor CD28 is the primary costimulatory receptor for initial T-cell expansion and survival and the positive costimulatory signals provided by CD28 lead to dramatic increase in IL-2 secretion and promote clustering of TCRs, which potentiate TCR signaling [14]. CD28 binds to B7-1 (CD80) and B7-2 (CD86), expressed exclusively on professional APCs, and this enhances T-cell proliferation by increasing the transcription of IL-2 and Bcl-xL [14]. Several other positive costimulatory molecules besides B7-1 and B7-2 exist that contribute to promote T cell functions and include inducible T-cell costimulator (ICOS: CD278), OX40 (CD134), 4-1BB (CD137), and CD40. In addition to the costimulatory molecules that promote T-cell activation other molecules exist that instead, regulate and inhibit T-cell activation. Herein, we review the role of inhibitory molecules that are expressed on cells or secreted following HIV-1 infection, and focus on their significance in HIV-associated immune inhibition. Our recent findings showed that HIV-1 exposed DCs gave rise to increased expression of inhibitory molecules on expanded T cells (Figure 1) and that these T cells had the ability to act in a contact-dependent manner on T cells present in their vicinity and suppressed their immune activation [2-4] (Figure 1). We also highlight the ensemble of repression factors and molecular pathways that facilitate the recruitment and differentiation of exhausted T cells in response to HIV-1 infection. The nature of the ensuing immune response depends on the initial stimuli and the binding amplitude of TCR-MHC-peptide complex formed during a given event of antigen presentation and subsequent engagement of positive or negative costimulatory molecules to their cognate receptors/ligands [15]. Chronic HIV infection reportedly induces expression of suppressor/inhibitory molecules that generate key negative signals that downregulate the ensuing T-cell responses.

### Negative costimulatory molecules

#### a) PD-1

**Figure 2:**
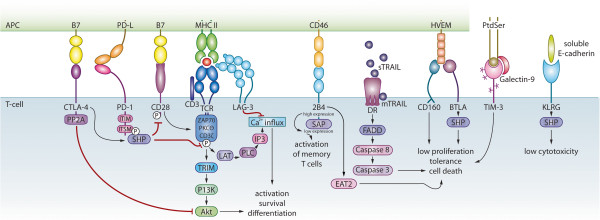
**Inhibitory signaling events at the DC-T cell interjunction leading to T-cell inhibition in HIV infection.** The inhibitory molecules expressed on APCs and T cells regulate the TCR-mediated signals. CTLA-4 and PD-1 recruit the key protein tyrosine kinases SHP-1 and SHP-2 leading to decreased IL-2 production and T-cell inhibition. CTLA-4 and PD-1 block CD28-mediated increase of glucose metabolism by interfering with Akt phosphorylation. PD-1 blocks the activation of phosphatidylinositol-3-kinase and CTLA-4 acting further downstream. LAG-3 induces high level of T-cell inhibition independent of other inhibitory molecules. LAG-3 functions by binding to the CD3/TCR complex where it inhibits CD3/TCR signaling and TCR-induced Ca^2+^-fluxes. 2B4-mediated CD8^+^ T-cell inhibition occurs via 2B4 binding to CD48 leading to recruitment of EAT2 adaptor molecule. TRAIL can interact with DR receptors to induce T-cell suppression without initiating apoptosis. Engagement of BTLA on T cells with HVEM inhibits TCR-mediated signaling via ITIM motifs and recruitment of SHP. Likewise CD160 also engages with the HVEM inhibiting the cell cycle functions of T-cell proliferation. Similarly, TIM-3-galectin9/phosphatidylserine and soluble E-cadherin-KLRG engagements could also lead to T-cell inhibition.

PD-1 (CD279) is a 50–55 kD glycoprotein that belongs to the CD28/B7 Ig superfamily. PD-1 expression can be induced on CD4^+^ and CD8^+^ T cells, natural killer cells (NK cells), T cells, B cells, and monocytes when these cells are activated [16,17]. The PD-1/PD-L pathway leads to the transduction of a negative immunoregulatory signal that antagonizes the TCR-CD28-mediated activation of phosphatidylinositol 3-kinase (PI3K), which reduce Akt phosphorylation and glucose metabolism resulting in inhibition of T-cell activation [18,19]**(**Figure 2). PD-L2 (B7-DC;CD273) and PD-L1 (B7-H1;CD274) are PD-1 ligands. PD-L2 expression is inducible on DCs and macrophages, whereas PD-L1 expression is constitutive on both professional and non-professional APCs [16,17,20,21]. Signaling via PD-1 occurs only when this receptor is engaged at the same time as TCR, which is in accordance with other CD28 family members. The cytoplasmic domain of PD-1 contains two tyrosine signaling motifs and both are phosphorylated following receptor engagement [18]. Phosphorylation of the second tyrosine, an immunoreceptor tyrosine-based inhibitory motif (ITSM), recruits SHP-2 and SHP-1 to the PD-1 cytoplasmic domain [18]. This initiates dephosphorylation of TCR proximal signaling molecules (e.g. ZAP70, PKCθ, and CD3ζ), leading to attenuation of the TCR/CD28 signaling cascade [18].

Accumulating lines of evidence suggest that the PD-1–PD-L1 pathway protects the vascular system from severe CD8^+^ T cell–mediated pathology during early systemic murine lymphocytic choriomeningitis virus (LCMV) infection. However, the association of PD-1 pathway with cytotoxic T lymphocyte (CTL) inhibition has opened up investigations on its potential negative role in HIV infection [4]. It has been shown that PD-1 expression is elevated on SIV-specific CD8^+^ T cells and *in vivo* blockade of the PD-1–PD-L pathway *in vivo* leads to increased T-cell proliferation, effector cytokine production, SIV-specific B-cell responses, and prolonged survival [19-22]. CD8^+^ T cells in HIV-infected individuals are reportedly dysfunctional with reduced proliferative capacity and effector functions [23]. In agreement with this notion, others showed that HIV disease severity i.e. viral load and declining CD4^+^ T-cell counts, correlated with level of both PD-1 expression on HIV-specific CD8^+^ T cells and percentage of cells expressing PD-1, providing a marker on CD8^+^ T cells that correlates with disease severity [23]. In addition, PD-1 expression on HIV-specific CD8^+^ T cells was markedly reduced in patients on ART, consistent with the notion that high antigen load drives PD-1 expression and functional exhaustion [23,24]. Importantly, HIV-exposed DCs induce T-cell inhibition via PD-1/cytotoxic T-lymphocyte antigen-4 (CTLA-4) signaling [6]. HIV exposure also leads to PD-L1 upregulation and B7-1/B7-2, and CD40 downregulation on myeloid DCs and this impairs DC functions, which correlates with disease progression in chronic HIV infection [25].

We and others have recently proposed that the PD-1 pathway could be manipulated for use in the treatment of persistent viral infections (PVIs), especially HIV-1 infection [5,21]. However, there is evidence suggesting that this pathway protects the vascular system from severe CD8^+^ T cell–mediated pathology during early systemic murine LCMV infection, indicating that immunopathological side effects might arise when interfering with the PD-1 pathway [19,20,26]. Accumulating evidence shows that HIV- and SIV-specific CTLs express high levels of PD-1, which contributes to the impaired proliferative T-cell responses [21,27,28]. The control of viral load in HIV and SIV infections correlates with reduced PD-1 expression on virus-specific CTLs, and PD-1 blockade results in enhanced HIV- or SIV-specific CTL proliferative responses [21,27,28]. Recent findings have extended the observation that T cells primed by HIV-pulsed DCs lead to expansion of T cells expressing multiple inhibitory molecules to include T-cell Ig mucin-containing domain-3 (TIM-3), lymphocyte activation gene-3 (LAG-3), and CTLA-4 besides PD-1 [2,4]. Further, HIV-specific CD8^+^ and CD4^+^ T cells that coexpress high levels of PD-1 and CD160 are more functionally impaired than cells with lower expression of these markers [29]. Hence, it is important to investigate the association of PD-1 with T-cell inhibition, especially in regards to the ability of virus-specific CTLs to kill infected cells. The mechanism underlying the regulation of PD-1 in activated and exhausted T cells is elusive. Recently, PD-1 upregulation via HIV Nef was shown to occur via a p38MAPK-dependent mechanism [30]. Several studies have confirmed that blockade of the STAT3, p38MAPK, NFATc, and PD-1 pathways results in enhanced T-cell proliferation *in vitro*[4,5,31]. Furthermore, the role of cytokine microenvironment, especially IL-2, IL-7, IL-15, and IL-21, in different tissues is emerging as one factor that can regulate PD-1/PD-L1 expression [32]. Importantly, transcriptional analyses of HIV-specific CD8^+^ T cells have shown that PD-1 could inhibit T-cell functions by upregulating basic leucine zipper transcription factor ATF-like (BATF) [33]. Hence, the impact of PD-1 is found to span across many signaling cascades and transcriptional factors, and is worth investigating.

#### b) CTLA-4

CTLA-4 (CD152) belongs to the costimulatory family of molecules and represents the Ig superfamily signaling via B7-1/B7-2 on APCs **(**Figure 2). It is homologous to CD28, but unlike CD28 it is a negative regulator of immune responses [34,35]. Unlike CD28, whose expression is constitutive, CTLA-4 expression is induced on T cells 24–48 hours after activation and CTLA-4 has greater affinity for both B7-1 and B7-2 than CD28. Following T-cell activation, the sequential action of Lck, Fyn, and RLK phosphorylates CTLA-4 and transports it to the cell surface. This negative regulator is constitutively expressed on CD4^+^CD25^+^FoxP3^+^ Tregs, which suppress autoimmunity and maintain peripheral tolerance, whereas other T-cell subsets express this factor only following activation [34,36]. Early studies demonstrated that CTLA-4 was upregulated on total CD4^+^ T cells of individuals with progressive HIV disease and that there was a negative correlation between CTLA-4 expression and CD4^+^ T-cell count [37]. Furthermore, studies in HIV-infected individuals at different stages of infection revealed that CTLA-4 also is selectively upregulated on HIV-specific CD4^+^ T cells in all categories of HIV-infected subjects besides long-term non-progressors (LTNPs) [38,39]. In contrast to PD-1, CTLA-4 is highly expressed on HIV-specific CD4^+^ T cells [25,40], but absent on HIV-specific CD8^+^ CTLs [38,39]. The HIV-specific CD4^+^ T cells with high CTLA-4 expression have impaired cytokine production and produce only IFN-γ, whereas cells with lower levels of CTLA-4 have the ability to secrete both IL-2 and IFN-γ [39]. *In vitro* blockade of CTLA-4 enhances HIV-specific CD4^+^ T cell functions, i.e. proliferation and IL-2 production [38], and decreases the susceptibility of these cells to become HIV infected [39].

#### c) TIM-3

TIM-3 belongs to the TIM family of molecules and TIM-1 through TIM-8 exist in mice, whereas humans express only TIM-1, TIM-3, and TIM-4 [41,42]. The TIM family members all have certain structural morphologies in common, i.e. an N-terminal immunoglobulin V domain, a mucin domain, and a transmembrane domain followed by a cytoplasmic tail [41-43]. TIM-3 binds to Gal-9, an S-type lectin, and induces T-cell tolerance or to phosphatidylserine and induces cell death [44,45] (Figure 2). Blocking the interaction between TIM-3 and Gal-9 resulted in exacerbated autoimmunity and abrogation of tolerance in experimental models [46]. Recent studies have established that TIM-3 also promotes CD8^+^ T-cell tolerance and myeloid-derived suppressor cell (MDSC) expansion in mice [47].

TIM-3 is expressed on Th1 cells and suppresses aggressive Th1 responses. TIM-3 expression is elevated on CD4^+^ and CD8^+^ T cells of HIV infected individuals [48-50]. We have shown that TIM-3 is expressed on T cells activated by HIV-pulsed DCs [2,4]. TIM-3 expressing T cells have poor proliferative abilities and dysfunctional cytokine responses, and *in vitro* blockade of TIM-3 results in improved proliferative ability for the HIV-specific T cells [50]. CD8^+^ T cell responses are crucial in controlling HIV-1 infection, and their role is emphasized by the impact the type of HLA class I alleles can have on progression to AIDS [51,52]. Most HIV-specific CD8^+^ T cells upregulate TIM-3 when interacting with their antigen epitope on MHC I molecule complexes. Quite the opposite occurs when HLA-B*27- and HLA-B*57-restricted HIV-specific CD8^+^ T cells encounter their epitopes, which leads to less upregulation of TIM-3 expression but higher production of granzyme B [53]. This clearly indicates that HIV-specific CD8^+^ CTLs restricted by specific haplotypes can evade immune suppression and continue to proliferate and kill virus infected cells. TIM-3 and PD-1 are coexpressed on both CD4^+^ and CD8^+^ T cells derived from individuals with chronic HIV [54] or HCV [48,55,56] infections and are associated with more severe CD8^+^ T-cell exhaustion [57]. Simultaneous blockade of PD-1 and TIM-3 pathways *in vivo* results in greater reversal of T-cell exhaustion and viral control compared to when only one of these pathways is blocked [57]. It has been shown that the STAT3/p38MAPK pathway contributes to upregulation of TIM-3 and therefore, it remains to be seen if blockade of TIM-3 upregulation contributes to improved functional abilities of Th1 cells in HIV infection.

#### d) LAG-3

LAG-3 (CD223) is a MHC II ligand belonging to the Ig superfamily expressed on activated and memory T cells, B cells and NK cells, and is upregulated by IL-2, IL-7 and IL-12. It is structurally homologous to the CD4 receptor, and is implicated in mediating T-cell suppression [58,59]. The LAG-3 induced T-cell suppression reportedly occurs via CD3/TCR complex-associated LAG-3 molecules inhibiting CD3/TCR signaling and TCR-induced Ca^2+^-fluxes [60]**(**Figure 2). LAG-3 induction requires a weaker stimulation compared to PD-1 ligation [61].

Studies in mice models have found that LAG-3 is capable of inducing T-cell suppression and that LAG-3 expression was linked to functional exhaustion of CD8^+^ T cells in persistent infections [62-64]. CD4^+^CD25^+^ nTregs express LAG-3 upon activation, and when this factor is deficient, i.e. in LAG-3^-/-^ mice, the cells exhibit impaired regulatory activity [60], which shows that LAG-3 contributes to the suppressor functions of Tregs. Furthermore, LAG-3 and PD-1 cooperate in the T-cell suppression and blockade of PD-1 and LAG-3 inhibitory receptor pathways improve T-cell responses in a synergistic manner [61]. However, not all data regarding LAG-3 points to a suppressive effect. For instance, a recent study failed to show the suppressive effects of LAG-3 [65]. LAG-3 levels are elevated in subjects with HIV infection [59] and our recent *in vitro* results are consistent with the notion that HIV exposure could increase LAG-3 expression and that this factor could play a negative role in HIV infection [2-4]. However, the functional relevance of LAG-3 in regulating T cell responses in HIV infection remains to be investigated further to establish if the elevated levels of this factor are part of the immune suppression seen in HIV infection.

#### e) CD160

CD160 is another member of the B7/CD28 family acting as a negative costimulatory receptor. It was originally identified as a MHC class I activating receptor on NK cells [64]. CD160 and BTLA binds both to the ligand HVEM expressed on APCs and activated T cells. Today, CD160 expression has been found on cytotoxic cells such as CD56^dim^CD16^–^ NK cells, NKT cells, γδT cells, CD8^+^CD28^–^ T cells, intraepithelial T cells, and a small subset of peripheral CD4^+^ and CD8^+^ T cells [66], and this receptor negatively regulates cell cycle [67]. Normally, CD160 is expressed on 5% of the CD4^+^ T cells, but a population of CD4^+^CD160^+^ cells can be fond in cutaneous inflammatory lesions [66,68]. CD160 expression is induced in a similar manner as CTLA-4 in T cells and mediates negative signaling [67]. When human CD4^+^ T cells are activated, they upregulate CD160 expression and when this receptor is cross-linked with HVEM this strongly inhibits CD4^+^ T-cell proliferation and cytokine production [69,70] (Figure 2). These findings clearly confirm CD160 as a negative regulator of CD4^+^ T-cell activation. The *ex vivo* expression level of CD160 is augmented in the lymphatic tissues derived from HIV-1-infected individuals during the acute stage of the disease [71]. In addition, CD160 expression is increased in acute and chronic HIV infections both on CD8^+^ T cells in general and on HIV-specific CD8^+^ T cells [28,71], which is in line with our recent observations *in vitro*[2,4]. Blockade of CD160 ligation with HVEM improves HIV-specific CD8^+^ T-cell proliferation and cytokine levels [29]. Recently, it has been reported that CD160^+^PD-1^+^CD8^+^ T cells define a subset at an advanced stage of immune exhaustion [29] and this underlines the importance of co-expression of inhibitory molecules in HIV-associated T-cell exhaustion.

#### f) BTLA

BTLA (CD272) is a negative costimulatory molecule belonging to the B7/CD28 family. BTLA is constitutively expressed at low levels on naïve B and T cells, macrophages, DCs, NKT cells, and NK cells [66]. It binds to its cognate ligand HVEM, a member of the TNFR superfamily expressed on APCs and Tregs [66]. BTLA expression is upregulated following T-cell activation. Similarly to CD160, BTLA has impairing effects on the cell cycle (Figure 2) [69] and inhibits TCR-mediated signaling via ITIM and ITSM motifs [72]. Engagement of BTLA on T cells with its ligand HVEM inhibits effector CD4^+^ T-cell functions [66,69,70]. Although BTLA has been proposed to be a negative regulator of T-cell activation, its potential inhibitory function is still inconclusive in HIV-1 infection. Our studies showed that BTLA upregulation was indistinctive on HIV-infected T cells *in vitro*[2,4] while others have reported that HIV-1 infection could downregulate BTLA on CD4^+^ and CD8^+^ T cells [73,74]. A recent finding demonstrated that HIV-1 could induce BTLA down-regulation on CD4^+^ T cells *in vitro* in an IFN-α dependent manner and this contributed to T-cell hyperactivation [73]. In agreement with this, dysregulation of B cells in HIV-1 infection has been associated with decreased BTLA expression on these cells in viremic individuals compared to aviremic individuals and healthy controls [1]. However, the functional significance of BTLA in HIV infection needs to be further evaluated.

#### g) 2B4

2B4 (CD244) belongs to the signaling lymphocyte activation molecule (SLAM) family whose members are implicated in the regulation of costimulation, cytokines, and cytotoxic activities [75]. This transmembrane protein is expressed by all NK cells, monocytes, basophils, eosinophils, γδ T cells, and memory CD8^+^ T cells [75]. CD48 is the cognate ligand of 2B4 and is expressed on NK cells [76]. 2B4 is an inhibitory receptor [77] regulating CD8^+^ T-cell functions and its expression could be a marker of CD8^+^ T-cell impairment [76]. Cross-linking of 2B4 with anti-2B4 mAb leads to NK-cell activation [76]. However, elevated 2B4 expression and relative paucity of signaling of 2B4’s intracellular adaptor molecule SAP promote an inhibitory function of 2B4 (Figure 2) [76,78]. Studies have shown that 2B4 expression on NK cells is increased in HIV-1 infected patients [79]. Further, the proportion of 2B4^+^CD8^+^ T cells is associated with immune activation of memory T cells, which increases with disease progression [80]. It is also clear that the ability to produce IFN-γ and cytotoxic activity of HIV-specific 2B4^+^CD8^+^ T cells is relatively lower compared to influenza-specific 2B4^+^CD8^+^ T cells in HIV infected individuals [81], and *in vitro* blockade of 2B4 increases the proliferative capacity of HIV-specific CD8^+^ T cells [82]. Moreover, downregulation of SAP in 2B4^+^CD8^+^ T cells upon HIV stimulation suggests an inhibitory role of 2B4^+^CD8^+^ T cells against constrained HIV epitopes, underlining the inability to control HIV during disease progression.

#### h) LILRB

Members of the leucocyte immunoglobulin-like receptor B (LILRB) family are expressed on B cells, mast cells, macrophages, monocytes, osteoclasts, NK cells and DCs [83,84] and are the human counterpart of the murine inhibitory molecule, PIR-B. Research has shown that LILRB1 can also be a T-cell factor that binds to HLA-A, HLA-B, HLA-F, HLA-G, and HCMV UL18 ligands [83,84]. DCs interaction with suppressor molecules on regulatory T cells rendered them tolerogenic by inducing upregulation of LILRB2 and LILRB4 [84]. High levels of LILRB1 and LILRB2 are observed during chronic HIV infection [85-87] and it has been shown that IL-10 upregulates LILRB2 in the monocytes of HIV-infected individuals, resulting in CD4^+^ T cell depletion [88]. However, LILRB1 and LILRB3 expression on circulating myeloid DCs of HIV elite controllers contributes to greater antigen-presenting potentials and their blockade abrogates the antigen-presenting properties of DCs [89]. This indicates that the regulatory functions of various members of the LILRB family are multifaceted.

#### i) TRAIL

TRAIL is a member of the TNF superfamily, and functions as a proapoptotic ligand [90]. The two biologically active forms of TRAIL, membrane-bound (mTRAIL) and soluble TRAIL (sTRAIL), are regulated by type I IFNs [91,92]. sTRAIL is secreted by leukocytes, including T cells, NK cells, DCs, monocytes, and macrophages [90,91,93]. TRAIL can interact with DR4 and DR5 receptors, capable of inducing apoptosis [93,94] and three other receptors that facilitate suppression without initiating apoptosis [93] (Figure 2). The elevated mTRAIL levels on T cells exposed to HIV-pulsed DCs [2,4] is intriguing because it can negatively regulate proliferation via mechanisms distinct from apoptosis [90]. Studies have shown that TRAIL is elevated in HIV-infected compared to uninfected subjects, and that when ART lowers the viral load dramatically, the TRAIL expression decreases [90]. Hence, TRAIL could be one potential inhibitory factor contributing to T-cell suppression in HIV infection.

#### j) KLRG1

KLRG1 is a member of the C-type lectin family of inhibitory receptors, which plays a unique but poorly characterized role in mediating T-cell exhaustion [95,96]. Soluble E-cadherin is the ligand for KLRG1. KLRG1 is expressed on a subset of CD4^+^ and CD8^+^ T cells, as well as on NK cells, and inhibits CD8^+^ T cell cytotoxicity and cytokine production [95,96] (Figure 2). KLRG1 is upregulated on virus-specific CD8^+^ T cells in response to repetitive antigenic stimulation in PVIs such as CMV and EBV [95,96]. The presence of the KLRG1 ligand, soluble E-cadherin, impairs the KLRG1^hi^ HIV-1–specific CD8^+^ T cells’ ability to respond by cytokine secretion upon antigenic stimulation and to inhibit viral replication [77]. Furthermore, KLRG1 is coexpresssed with other inhibitory receptors, i.e. PD-1, CD160, and 2B4, on exhausted HCV-specific CD8^+^ T cells [77]. Of note, a recent study showed that knockout of KLRG1 in mice did not have an apparent effect on the phenotype, suggesting that KLRG1 might not contribute significantly to T cell exhaustion during HIV infection [97].

### Transcriptional factors and pathways

Recent lines of evidence have highlighted the importance of inhibitory molecules and related pathways of T-cell exhaustion. However, the underlying transcriptional mechanisms remain for the most part elusive. In addition to the multiple inhibitory receptors that are involved in T-cell exhaustion, persistent changes in transcription patterns are observed when comparing the molecular signatures of exhausted T cells to functional T cells. These changes include altered expression of transcription factors, changes in signal transduction, and down-regulation of key metabolic genes [2].

#### a) BLIMP-1

BLIMP-1 (designated PRDI-BF1 in humans), a zinc finger-containing evolutionarily conserved transcriptional repressor encoded by *PRDM1*, is an important factor implicated in the generation of terminally differentiated plasma cells [98]. BLIMP-1 has also been reported to be a master regulator of terminal differentiation of CD8^+^ T-cells [99]. Recently, it has been shown that its elevated expression directly correlates with the upregulation of an array of cell surface inhibitory molecules in chronic viral infection [63] (Figure 1). BLIMP-1 attenuates T-cell proliferation and CD4^+^ Treg functions, and its expression is reportedly enhanced in antigen-experienced T cells [100-102]. BLIMP-1 promotes the overexpression of inhibitory receptors and also suppresses key molecules involved in normal memory CD8^+^ T-cell differentiation, such as IL-7 receptor and CD62L [63]. Moreover, coexpression of FoxP3 and BLIMP-1 could be vital for suppressor functions as FoxP3 reportedly leads to activation of BLIMP-1 in antigen-exposed T-cells [102]. Intriguingly, high BLIMP-1 expression correlates with increased PD-1, CTLA-4, and CD160 expression in chronic HIV infection [63]. During acute infection, smaller amounts of BLIMP-1 are associated with terminal differentiation of effector FoxP3^+^CD8^+^ T cells [102], whereas high BLIMP-1 expression during chronic infection promotes upregulation of inhibitory receptors including PD-1, LAG-3, CD160 and 2B4, resulting in exhausted CD8^+^ T cells [63]. While lack of BLIMP-1 gives defective cytolytic function in virus-specific CD8^+^ T cells and low expression of KLRG1 [77], the potential role of BLIMP-1 in the upregulation of multiple inhibitory molecules is clear in chronic viral infections, especially in LCMV and HIV-1 infection [2,4,63,103]. We have demonstrated that BLIMP-1 is induced in CD4^+^ T cells stimulated by HIV-exposed DCs [2,4] and recent lines of evidence points to the existence of a novel miR-9/BLIMP-1/IL-2 axis that is compromised in progressive HIV disease but not in LTNPs [104,105]. BLIMP-1 is upregulated in CD4^+^ T cells via TCR stimulation and IL-2 and this is regulated by miR-9 levels. The upregulation of miR-9 induces BLIMP-1 repression, leading to restoration of IL-2 secretion by CD4^+^ T cells, which occurs by reduced binding potential of BLIMP-1 to the *il-2* promoter [104,105].

#### b) FoxP3

FoxP3 regulates CD4^+^T-cell activation and FoxP3 expression is elevated in T cells upon stimulation leading to suppressive functions (Figure 1) [106] and HIV and SIV infections can give rise to FoxP3 expression in T cells [106-111]. Recent findings associated increased FoxP3 expression with the onset of T cell dysfunction in HIV/AIDS [112]. Interestingly, high CTLA-4 expression on Tregs depends on FoxP3 along with NFAT [111,113]. The elevated expression of FoxP3 and BLIMP-1 in T cells primed with HIV-pulsed DCs suggests a potential direct role of FoxP3 in controlling BLIMP-1 expression in antigen-exposed T cells [2,4]. This is consistent with prior observations from a genome-wide investigation, which showed that BLIMP-1 is directly activated by FoxP3, adding a key dimension to the notion that BLIMP-1 is necessary for accurate function of suppressor T cells [101].

#### c) T-bet

T-bet, encoded by *Tbx21* gene, is the key regulator of the Th1 phenotype differentiation system. It induces the synthesis of IFN-γ and regulates the expression of chemokines and chemokine to orchestrate Th1 cell differentiation. Expression of T-bet together with granzyme A and B, granulysin, and perforin has been assessed in HIV-specific CD8^+^ T cells derived from elite controllers, progressors, and ART treated individuals [114]. Interestingly, the HIV-specific CD8^+^ T cells from elite controllers had greater capacity for granzyme B and perforin expression relative to the other groups [114] and level of T-bet expression in HIV-specific CD8^+^ T cells correlated with granzyme B and perforin levels [114]. Hence, it has been suggested that T-bet can regulate the expression of perforin and granzyme B by binding to the promoter regions of these genes [115-117]. In chronic LCMV, T-bet directly represses the gene promotor for PD-1 in a site-specific manner, which leads to lower expression of PD-1 and other inhibitory receptors [117,118]. It was also demonstrated that genetic ablation of T-bet leads to exacerbation of CD8^+^ T-cell exhaustion and increase of viral load [118]. BLIMP-1 and T-bet seem to have similar roles in promoting the effector function and terminal differentiation of CD8^+^ T cells during acute infection [102,117]. High T-bet expression promotes terminally differentiated CD127^lo^KLRG-1^hi^ effector CD8^+^ T cells and sustains functional virus-specific CD8^+^ T-cell responses [117]. Exhausted CD8^+^ T cells have downmodulated T-bet levels due to persistent antigenic stimulation results in exhausted CD8^+^ T cells [117]. Whereas the exact mechanism of repression of T-bet expression is unknown, deficiency of T-bet leads to attenuated BLIMP-1 expression in NK cells [119], and the same effect may be expected in the CD8^+^ cell, as BLIMP-1 and T-bet-deficient CD8^+^ T cells show similar differentiation phenotypes [102,120,121].

#### d) BATF

BATF has been identified as a negative regulator of AP-1 by forming dimers with c-Jun [122], which inhibit canonical AP-1-mediated transcription, and this contributes to T cell exhaustion [123]. BATF regulates effector CD8^+^ T-cell differentiation via Sirt1 expression [124,125]. PD-1 ligation can inhibit T cell functions by enhancing BATF expression and this has been documented in HIV-specific CD8^+^ T cells derived from infected individuals [33]. It has also been shown that BATF overexpression in activated primary human T cells impairs T-cell proliferation and IL-2 production, whereas silencing BATF expression in HIV-specific T cells increases their proliferation, as well as IFN-γ and IL-2 production [33,124], confirming that BATF plays a role in T cell dysfunction during HIV infection. In addition, BATF is also required for the differentiation of IL17-producing Th17 cells, which coordinate inflammatory responses in host defense [125].

#### e) p38MAPK/STAT3

The STAT3 pathway can be activated either by IL-10 and IL-6 cytokines or by growth factors such as VEGF, TGF-β, G-CSF, PDGF, EGF and MAPkinases [126,127]. Recently, we have reported that p38MAPK/STAT3 pathways were involved in HIV-1 mediated upregulation of inhibitory receptors CTLA-4, TRAIL, TIM-3, LAG-3, CD160 and transcription factors BLIMP-1, DTX1, and FoxP3, as their blockade abolished expression of inhibitory molecules and restored T-cell proliferation *in vitro*[4]. Specifically, it has been found that HIV Nef mediates PD-1 upregulation via a p38MAPK-dependent mechanism [30].

#### g) NFATc1 and DTX1

An impaired NFAT nuclear translocation is observed in exhausted CD8^+^ T cells during chronic HIV and LCMV infections [128,129]. The nuclear translocation of NFATc1 (NFAT2) was more efficient in HIV-specific CD8^+^ T cells derived from LTNPs relative to individuals with disease progression [130]. Inhibition of calcineurin or NFAT leads to sharp reduction in PD-1 expression suggesting a regulatory role for calcineurin/NFAT signaling pathway [129,130]. However, it remains to be investigated how altered nuclear translocation of NFATc1 and PD-1 expression are associated with exhausted T cells. DTX1 is a transcription target of NFAT, and upregulation of DTX1 inhibits T-cell activation by both E3-dependent and E3-independent mechanisms [131]. Recently, we reported that HIV-1 induced increased expression of DTX1 mRNA in the T cells primed by HIV-1 exposed DCs, which correlated with increased NFAT mRNA [4]. We also found that inhibition of NFAT decreased DTX1 and PD-1 mRNA and protein expression.

#### h) Miscellaneous pathways

##### FOxO3a

FOxO3a is a transcription factor constitutively expressed in hematopoietic cells that can promote the transcription of certain proapoptotic target genes e.g. Bim, FasL, and TRAIL [132]. HIV TAT-induced FOXO3a in association with these factors reportedly play a major role in mediating the apoptosis of HIV-1-infected human CD4^+^ T cells [133]. A study showed that FOxO3a/TRAIL signaling has a direct role in the persistence of memory B cells during HIV infection [134]. Transcriptional activity of FOxO3a and expression of TRAIL have been found to be higher in aviremic treated individuals compared to elite controllers and uninfected individuals and have been attributed to low survival rates of memory B cells [134].

##### Socs3

Socs3 has recently been shown to facilitate T-cell exhaustion in chronic infections [135]. LCMV-specific T cells in chronic infection express higher levels of Socs3, whereas Socs3 deficiency leads to enhanced T cell functions. Interestingly, IL-7 treatment results in decreased levels of Socs3 and reinvigorates the immune response to chronic virus infection [135,136]. Hence, downregulation of Socs3 using IL-7 is likely to contribute to improve T-cell functions. The role of Socs3 in HIV-1 infection remains to be investigated.

##### Hippo pathway

The Hippo pathway is a highly conserved developmental system, which directly controls terminal differentiation of multiple cell types in invertebrates and vertebrates [137]. Recently, it was shown that activation of the Hippo pathway by CTLA-4 regulates the expression of BLIMP-1 in CD8^+^ T cells [121]. The CTLA-4/Hippo pathway/BLIMP-1 system may link terminal differentiation of CD8^+^ T cells [121]. However, the precise role of the association of CTLA-4/Hippo/BLIMP-1 network in HIV infection remains to be elucidated.

### Immunoregulatory cytokines and enzymes

#### a) IDO

IDO is an intracellular enzyme that catalyses the catabolism of tryptophan. IFN-γ is the primary inducer of IDO while other factors such as TNF-α, TNF-β and lipopolysaccharide can induce IDO to a limited extent [138,139]. In 2002, it became evident that CTLA-4 ligation to B7 resulted in the induction of an IDO^+^ immunosuppressive DC phenotype (Figure 1) [40]. Subsequently, CTLA-4/B7-mediated IDO induction was observed in myeloid DCs, pDCs, and MDDCs [140,141]. Increased IDO activity leads to apoptosis of effector T cells and induction of Tregs thereby dampening an active immune response [142]. These Tregs participate in a positive feedback loop via CTLA-4 engagement of B7 molecules, which stimulate increased IFN-γ production from APCs and subsequent enhancement of IDO activity [142]. The reduction of plasma concentration of tryptophan in HIV-1 patients was first reported in 1988 [143] and thereafter it has been shown that HIV infection could result in increased IDO activity [144]. It is becoming clear that TGF-β1 signaling through a PI3K-dependent or a SMAD-independent pathway can induce Fyn-dependent phosphorylation of IDO ITIMs [145], which leads to activation of noncanonical NF-kB to activate IDO signaling [145]. Therefore, approaches blocking the IDO pathway may be a potential strategy to improve T-cell functions in HIV-infected patients.

#### b) IL-10

IL-10 was first recognized for its ability to inhibit activation of T cells, B cells, monocytes, and macrophages, and also to terminate inflammatory responses [146,147]. IL-10 is produced by CD4^+^ T cells, including Tregs, CD8^+^ T cells, DCs, macrophages, and B cells [146,147]. Increase in IL-10 levels has been reported in PVIs, including HIV and HCV [148]. Interestingly, it has been shown that IL-10 and PD-L1 pathways work in synergy to suppress T-cell activation during persistent LCMV infection, and that blockade of both IL-10 and PD-L1 more effectively restores antiviral T-cell responses than blockade of either one alone [149]. The PD-1–induced IL-10 production by monocytes could impair CD4^+^ T cell activation during HIV infection [150]. Furthermore, the levels of serum IL-10 and IL-10 mRNA in PBMCs are reported to increase with HIV disease progression [151] and IL-10 reversibly inhibits virus-specific T cells [152]. Blockade of IL-10 restored Env- specific T-cell proliferative responses to a high degree [153], although, this ability was eventually lost during advanced HIV disease [152].

#### c) TGF-β

TGF-β is an immunoregulatory cytokine that is implicated in controlling immune responses and maintaining immune homeostasis by affecting proliferation, differentiation, and survival of multiple immune cell lineages [154]. Upregulation of TGF-β and IL-10 is associated with disease progression in HIV-1-infected individuals [155]. TGF-β upregulates CTLA-4 expression and suppresses IL-2 production and T cell proliferation [156]. Moreover, it has been reported that TGF-β and IL-10 production by HIV-specific CD8^+^ T cells regulates CTLA-4 signaling on CD4^+^ T cells [155]. Noteworthy is that blockade of TGF-β did not improve control of chronic LCMV infection [157,158], which suggests that blocking this factor alone might not have any effect on the control of HIV-1 infection.

## Conclusion

Our improved understanding of the T-cell costimulation and coinhibition pathways attained over the past decade has given plenty of evidence on the key roles played by these molecules in immune homeostasis. However, numerous infectious agents and tumors escape from host immune surveillance by efficiently upregulating coinhibitory signals. It is now clear that coexpression of multiple distinct inhibitory receptors is associated with greater T cell exhaustion and rapid HIV disease progression. It has also been established by researchers that T-cell inhibition results from progressive sequential accumulation of a broad array of inhibitory molecules in HIV infection. Hence, measures to understand their contribution to T-cell suppression and target the molecular and biochemical signaling networks that converge to inhibit T-cell activation need to be further investigated. Our recent findings have shown that inhibitory molecules are under the control of diverse pathways, i.e. PD-1 is upregulated by both p38MAPK/STAT3 and NFAT pathways, whereas CTLA-4, TRAIL, LAG-3, CD160 and TIM-3 are regulated by p38MAPK/STAT3. Of interest to further elucidate is for instance how HIV-1 exploits DCs, inducing them to secrete retinoic acid, which is believed to trigger the differentiation of tolerogenic T cells. Further, it is clear that inhibitory receptors are potential targets of therapeutics in HIV infection and therefore it is important to decode the molecular signatures of T-cell suppression as this might open up for new drugs targeting inhibitory molecules, transcriptional repressors and pathways in HIV infected individuals.

Although there is no experimental evidence, one approach we suggest is to block inhibitory molecules, especially PD-1/PD-L1, to amplify antiviral T-cell functions to a level sufficient enough to purge latent viral reservoirs. Certain key questions still remain to be answered; will the therapeutic use of targeting inhibitory molecules in HIV be toxic to HIV-infected individuals? What will be the magnitude of damage caused to the house-keeping functions of the coinhibitory molecules targeted? Will this targeting bring any additional benefit to ART-treated subjects? Exploring these areas may be necessary to ensure successful response of chronic HIV infected patients to anti-inhibitory molecular therapeutics. Therefore, the prime objective would be to facilitate complete functional restoration of T-cell functions, which may rely on combination therapies targeting diverse sets of host cellular factors at different stages of HIV infection. Given the emergence of a wider network of inhibitory molecules in HIV infection, additional studies may be required to investigate the molecular targets associated with restoration of T-cell functions to increase longevity and quality of life of HIV-infected individuals.Exhausted T cells : Memory T cells that assume a state of unresponsiveness following activation by certain viral antigens that are noticeable during subsequent antigenic stimulation [159].1. Natural Tregs (nTregs): CD4 ^+^ CD25 ^+^ CD127^low^ phenotype cells that develop in the thymus. nTregs are CTLA-4 ^+^ GITR ^+^ Foxp3^+^. They facilitate auto reactive T-cell suppression by contact, cytolytic mechanisms, or by TGF-β. nTregs expand *in vivo* following TCR/CD28 stimulation and by expressing receptors for IL-2.; 2. Induced Tregs (iTreg): Non-regulatory CD4^+^ T cells, which acquire CD25 (IL-2Rα) expression outside of the thymus. **a) Tr1:** CD4 ^+^ CD25- phenotype that develops in the periphery. Tr1 cells are marked by CD45RB^low^Foxp3- and mediate suppression via IL-10. Tr1 cells expand following CD3 signaling leading to secretion of IL-10 and retinoic acid. **b) Tr3:** CD4 ^+^ CD25^+^, develop in the periphery under the influence of TGF-β from CD4^+^ CD25-Treg precursors. Tr3 cells are marked by CD25^low-variable^CD45RB^low^Foxp3^+^ and mediate suppression via TGF-β. Expand following CD3 signaling leading to secretion of TGF-β.; Suppressor T cells: T cells that arise following priming by HIV-exposed DCs. Suppressor T cells reportedly express numerous molecules that could facilitate T-cell inhibition in a contact-dependent manner [2-4].

## Abbreviations

AIDS: Acquired immunodeficiency syndrome; APC: Antigen-presenting cell; ART: Antiretroviral treatment; BATF: Basic leucine zipper transcription factor ATF-like; Bcl-xL: B-cell lymphoma-extra large; BLIMP-1: B-lymphocyte-induced maturation protein; BTLA: B and T-lymphocyte attenuator; CMV: Cytomegalovirus; CTLA-4: Cytotoxic T-lymphocyte antigen-4; CTL: Cytotoxic T lymphocyte; DC: Dendritic cell; DTX1: Deltex homolog 1 protein; EAT2: Ewing’s sarcoma-Fli1-activated transcript 2; EBV: Epstein-barr virus; EGF: Epidermal growth factor; FoxP3: Fork-head transcription factor P3; Gal-9: Galectin-9; G-CSF: Granulocyte colony stimulating factor; GITR: Glucocorticoid-induced tumor necrosis factor receptor; HBV: Hepatitis B virus; HCV: Hepatitis C virus; HIV-1: Human immunodeficiency virus type-1; HLA: Human leukocyte antigen; HSV: Herpes simplex virus; ICOS: Inducible T-cell costimulator; IDO: Indoleamine 2, 3-dioxygenase; IFN-γ: Interferon-gamma; IL-6: Interleukin-6; IL-7: Interleukin-7; IL-10: Interleukin-10; ITIM: Immunoreceptor tyrosine-based inhibitory motif; iTregs: Inducible regulatory T cells; ITSM: IT–based switch motif; JAK: Janus Kinase; KLRG1: Killer cell lectin-like receptor G1; LAG-3: Lymphocyte activation gene-3; LCK: Lymphocyte cell kinase; LCMV: Lymphocytic choriomeningitis virus; LILR: Leukocyte Ig-like receptor; LILRB: LIL receptor B; LILRB1: LILRB member 1; LPS: Lipopolysaccharide; LTNP: Long-term non-progressor; 1-MT: 1-methyltryptophan; mAb: Monoclonal antibody; mDC: myeloid dendritic cell; MDDC: Monocyte-derived dendritic cell; miR-9: MicroRNA-9; mTRAIL: Membrane-bound tumor-necrosis factor-related apoptosis-inducing ligand; MDSC: Myeloid-derived suppressor cell; NAD+: Nicotinamide adenine dinucleotide; NFATc: Nuclear factor associated with transcription; NK: Natural killer cell; NKT: NK T cell; nTregs: Natural regulatory T cells; mRNA: Messenger RNA; p38MAPK: p38 mitogen-activated protein kinase; PBMC: Peripheral blood mononuclear cell; PD-1: Programmed death-1; pDC: Plasmacytoid DC; PDGF: Platelet-derived growth factor; PI3K: Phosphatidylinositol 3-kinase; PIR-B: Paired Ig-like receptor B; PKCθ: Protein kinase C theta; PVI: Persistent viral infection; PRDM1: Positive regulatory domain 1-binding factor; RLK: Resting lymphocyte kinase; SAP: SLAM-associated protein; siRNA: Small interfering RNA; SIV: Simian immunodeficiency virus; Socs3: Suppressor of cytokine signaling 3; STAT3: Signal transducer and activator of transcription 3; TCR: T-cell receptor; TGF-β1: Transforming growth factor-beta1; TIM-3: T-cell immunoglobulin mucin-containing domain-3; TNF: Tumor necrosis factor; TRAIL: TNF-related apoptosis-inducing ligand; Treg: Regulatory T cell; VEGF: Vascular endothelial growth factor; ZAP-70: Zeta-chain-associated protein kinase-70

## Competing interests

The authors declare that they have no competing interests.

## Authors’ contributions

ML, EMS, KFC, RE, AS and VV generated the initial manuscript draft and the figures; VR, RA, RE, AK, MB, RR and ML contributed to writing and jointly developed the article to its final form. All authors read and approved the final manuscript.
